# Supplementary material for: Single-Dose SDA-Rich Echium Oil Increases Plasma EPA, DPAn3, and DHA Concentrations
Source: Nutrients. 2019 Oct 2;11(10):2346. doi: 10.3390/nu11102346 (PMC6835614; doi:10.3390/nu11102346)
Supplement: Supplementary file 1 [file nutrients-11-02346-s001.pdf]

Supplementary Information

Single-dose SDA-rich Echium oil increases plasma EPA, DPAn3 and DHA concentrations

Supplementary Information on Recruitment, screening and selection of participants
2 Supplementary tables (table S1-S2)

Recruitment, screening and selection of participants

Participants were recruited from the general population in Hannover, Germany by advertisements. Subjects were pre-selected via screening questionnaires according to the following inclusion criteria: Male sex, age between 20 and 40 years, body mass index (BMI) between 20 and 27 kg/m², mixed diet with low meat and fish consumption. Exclusion criteria were defined as followed: Smoking, serum triglyceride (TG) levels ≥150 mg/dl (≥1.7 mmol/l); serum total cholesterol levels ≥200 mg/dl (≥5.2 mmol/l); a relative amount of ΣEPA+DHA in red blood cells ≤3 and ≥6%, intake of fish (>2 times per week) as well as addiction to alcohol, drugs and/or medications and diseases: chronic diseases (e.g. malignant tumors, manifest cardiovascular disease, insulin-dependent type 1 and 2 diabetes, severe renal or liver diseases); chronic gastrointestinal disorders (especially small intestine, pancreas, liver) as well as prior gastrointestinal surgical procedures (e.g. gastrectomy); hormonal disorders (e.g. Cushing´s syndrome and untreated hyperthyroidism); uncontrolled hypertension; blood coagulation disorders and intake of coagulation-inhibiting drugs; periodic intake of laxatives; intake of anti-inflammatory drugs (incl. acetylsalicylic acid); intake of lipid lowering drugs or supplements during the last 3 months before baseline examination. Inclusion and exclusion criteria were assessed via the screening questionnaire. The pre-selected subjects were invited to a screening examination to collect fasting blood for the analysis of serum lipid levels, liver enzymes and fatty acid patterns in blood cells. Serum triglyceride and total cholesterol were analyzed in the LADR laboratory (Laborärztliche Arbeitsgemeinschaft für Diagnostik und Rationalisierung e.V.), Hannover, Germany. For analysis of fatty acids in blood cells, the cell sediment after centrifugation and removal of plasma was reconstituted in PBS to the initial blood volume, transferred into 1.5 mL Eppendorf tubes and immediately frozen and stored at -80 °C until extraction and analysis. Lipids were extracted from 50 µL diluted blood cells using MTBE/MeOH and concentrations of fatty acids were determined by by means of gas chromatography with flame ionization detection (GC-FID) following (trans-)esterification to fatty acid methyl esters (FAMEs) as described (1) using methyl pentacosanoate (C25:0 methyl ester) as internal standard (IS) for quantification.

Table S1: Daily energy, macronutrient and fatty acid intake of study participants during the whole period of the standardized nutrition (Day 1, 2 and 3) **(A)** and energy, macronutrient and fatty acid intake of meals at Day 1 of the standardized nutrition **(B)**.

**A)**	**Day 1**		**Day 2**		**Day 3**	
**Portion size**	**small**	**large**	**small**	**large**	**small**	**large**
**Energy intake (kcal)**[a]	2924	3152	2687	2946	2907	3179
**Carbohydrates (g)**[a]	337	378	321	375	335	375
**Protein (g)**[a]	122	128	103	110	125	136
**Total fat intake (g)**[a]	82.0	82.3	106	108	85.33	85.32
**SFA (g)**[a]	37.06	35.31	40.88	38.55	38.50	37.34
**MUFA (g)**[a]	17.49	16.64	20.87	20.06	18.05	17.67
**PUFA (g)**[a]	3.98	4.00	10.20	10.16	4.86	5.32
**LA (g)**[b]	3.25	3.27	9.49	9.45	4.00	4.36
**ALA (g)**[b]	0.53	0.52	0.51	0.49	0.67	0.74
**ARA (g)**[b]	0.10	0.11	0.12	0.12	0.11	0.11
**EPA (g)**[b]	0.03	0.03	0.03	0.03	0.03	0.03
**DPAn3 (g)**[b]	< 0.01	0.02	< 0.01	0.02	0.01	0.02
**DHA (g)**[b]	< 0.01	< 0.01	< 0.01	< 0.01	< 0.01	< 0.01

Levels are shown at day 1, 2 and 3 of standardized nutrition for small and large portion size.

**B)**	**Breakfast**	**Lunch**	**Snack**		**Dinner**	
**Portion size**	**small / large**	**small / large**	**small**	**large**	**small**	**large**
**Energy intake (kcal)**[a]	900	919	223	319	882	1014
**Carbohydrates (g)**[a]	95.9	125	33.3	48.1	83.3	109
**Protein (g)**[a]	34.8	37.7	8.00	10.0	41.2	45.3
**Total fat intake (g)**[a]	36.6	6.80	3.78	4.10	34.9	35.1
**SFA (g)**[a]	17.82	2.71	2.16		14.37	12.62
**MUFA (g)**[a]	8.68	1.24	1.07		6.49	5.64
**PUFA (g)**[a]	1.86	0.33	0.12		1.67	1.69
**LA (g)**[b]	1.54	0.24	0.09		1.38	1.40
**ALA (g)**[b]	0.22	0.07	0.02		0.21	0.20
**ARA (g)**[b]	0.05	0.01	< 0.01		0.04	0.04
**EPA (g)**[b]	0.02	< 0.01	< 0.01		0.01	0.01
**DPAn3 (g)**[b]	< 0.01	< 0.01	< 0.01		< 0.01	0.01
**DHA (g)**[b]	< 0.01	< 0.01	< 0.01		< 0.01	< 0.01

Levels are shown for breakfast, lunch, snack and dinner of standardized nutrition for small and large portion size.

ARA: arachidonic acid; ALA: α-linolenic acid; EPA: eicosapentaenoic acid; DHA: docosahexaenoic acid; DPAn3: docosapentaenoic acid; LA: linoleic acid; MUFA: monounsaturated fatty acids; PUFA: polyunsaturated fatty acids; SFA: saturated fatty acids.

[a] Energy, carbohydrate and protein intake were calculated with PRODI®

[b] Total fat, SFA, MUFA and PUFA LA, ALA, AA, EPA, DPAn3 and DHA intake were calculated from own analyses of meals that were provided by the Institute of Food Science and Human Nutrition

<u>Table S2:</u> Fatty acid profile in plasma after single-dose ingestion of echium oil.

		t0	t2	t4	t6	t8	t24	t48	t72
		**mean ± SD**	**mean ± SD**	**mean ± SD**	**mean ± SD**	**mean ± SD**	**mean ± SD**	**mean ± SD**	**mean ± SD**
**C10:0**	µg/mL	<LLOQ	<LLOQ	<LLOQ	<LLOQ	<LLOQ	<LLOQ	<LLOQ	<LLOQ
	% of total FA	<LLOQ	<LLOQ	<LLOQ	<LLOQ	<LLOQ	<LLOQ	<LLOQ	<LLOQ
**C11:0**	µg/mL	<LLOQ	<LLOQ	<LLOQ	<LLOQ	<LLOQ	<LLOQ	<LLOQ	<LLOQ
	% of total FA	<LLOQ	<LLOQ	<LLOQ	<LLOQ	<LLOQ	<LLOQ	<LLOQ	<LLOQ
**C13:0**	µg/mL	<LLOQ	<LLOQ	<LLOQ	<LLOQ	<LLOQ	<LLOQ	<LLOQ	<LLOQ
	% of total FA	<LLOQ	<LLOQ	<LLOQ	<LLOQ	<LLOQ	<LLOQ	<LLOQ	<LLOQ
**C14:0**	µg/mL	34.4 ± 14.3	48.0 ± 28.0	52.6 ± 27.5	67.0 ± 40.0	39.0 ± 17.1	26.7 ± 14.1	33.9 ± 11.5	32.1 ± 12.8
	% of total FA	1.16 ± 0.29	1.55 ± 0.53	1.54 ± 0.40	1.74 ± 0.56	1.19 ± 0.31	1.01 ± 0.41	1.51 ± 0.31	1.09 ± 0.29
**C14:1n5**	µg/mL	1.82 ± 0.90	2.82 ± 1.84	3.43 ± 1.92	4.88 ± 3.19	1.88 ± 0.96	1.14 ± 0.70	1.63 ± 0.74	1.70 ± 1.09
	% of total FA	0.06 ± 0.02	0.09 ± 0.04	0.10 ± 0.03	0.12 ± 0.05	0.06 ± 0.02	0.04 ± 0.02	0.07 ± 0.02	0.05 ± 0.03
**C15:0**	µg/mL	11.1 ± 3.32	15.2 ± 5.53	14.1 ± 3.78	12.4 ± 5.12	15.0 ± 5.22	10.1 ± 3.25	10.2 ± 2.44	12.6 ± 2.58
	% of total FA	0.39 ± 0.12	0.52 ± 0.13	0.44 ± 0.07	0.34 ± 0.06	0.48 ± 0.15	0.40 ± 0.13	0.47 ± 0.09	0.44 ± 0.09
**C15:1n5**	µg/mL	<LLOQ	<LLOQ	<LLOQ	<LLOQ	<LLOQ	<LLOQ	<LLOQ	<LLOQ
	% of total FA	<LLOQ	<LLOQ	<LLOQ	<LLOQ	<LLOQ	<LLOQ	<LLOQ	<LLOQ
**C16:0**	µg/mL	656 ± 188	650 ± 242	731 ± 266	835 ± 272	682 ± 185	569 ± 156	502 ± 115	630 ± 141
	% of total FA	22.5 ± 1.64	21.9 ± 1.95	22.1 ± 2.04	23.0 ± 1.49	21.4 ± 1.58	21.9 ± 1.67	22.8 ± 1.05	21.7 ± 1.38
**C16:1n7**	µg/mL	73.2 ± 34.2	68.3 ± 32.7	68.7 ± 31.5	72.1 ± 30.2	60.1 ± 22.7	52.2 ± 22.6	48.4 ± 17.2	58.2 ± 17.5
	% of total FA	2.44 ± 0.66	2.25 ± 0.60	2.04 ± 0.53	1.96 ± 0.51	1.86 ± 0.52	1.96 ± 0.52	2.16 ± 0.45	1.99 ± 0.36
**C17:0**	µg/mL	8.28 ± 1.37	9.67 ± 2.53	11.0 ± 3.20	12.5 ± 3.93	10.2 ± 2.93	8.27 ± 1.56	7.34 ± 1.50	10.2 ± 2.22
	% of total FA	0.29 ± 0.04	0.34 ± 0.04	0.34 ± 0.04	0.35 ± 0.04	0.32 ± 0.05	0.32 ± 0.04	0.34 ± 0.03	0.35 ± 0.04
**C17:1n8**	µg/mL	<LLOQ	<LLOQ	<LLOQ	<LLOQ	<LLOQ	<LLOQ	<LLOQ	<LLOQ
	% of total FA	<LLOQ	<LLOQ	<LLOQ	<LLOQ	<LLOQ	<LLOQ	<LLOQ	<LLOQ
**C18:0**	µg/mL	200 ± 46.3	214 ± 58.7	250 ± 73.8	273 ± 73.0	247 ± 52.4	198 ± 43.1	160 ± 32.8	229 ± 41.2
	% of total FA	6.94 ± 0.58	7.45 ± 0.53	7.70 ± 0.57	7.64 ± 0.52	7.88 ± 0.67	7.68 ± 0.68	7.30 ± 0.49	7.96 ± 0.46

**C18:1n9**	μg/mL	701	±	227	691	±	259	763	±	278	791	±	277	620	±	170	529	±	144	423	±	102	562	±	106
	% of total FA	23.9	±	2.66	23.4	±	2.66	23.0	±	2.44	21.6	±	2.16	19.4	±	1.87	20.3	±	1.65	19.2	±	1.94	19.5	±	1.57
**C18:1n7**	μg/mL	64.9	±	21.2	67.1	±	25.4	70.8	±	24.4	74.2	±	22.8	61.4	±	15.6	53.9	±	14.5	44.3	±	11.1	60.7	±	12.0
	% of total FA	2.22	±	0.25	2.26	±	0.26	2.15	±	0.25	2.05	±	0.23	1.93	±	0.18	2.07	±	0.14	2.01	±	0.20	2.11	±	0.17
**C18:2n6**	μg/mL	706	±	120	667	±	146	731	±	145	797	±	155	790	±	140	645	±	117	572	±	103	733	±	121
	% of total FA	24.9	±	3.28	23.6	±	3.20	23.1	±	3.08	22.8	±	3.33	25.4	±	2.80	25.3	±	2.41	26.2	±	1.94	25.6	±	1.97
**C18:3n6**	μg/mL	11.0	±	4.69	20.9	±	11.3	28.7	±	19.5	51.7	±	27.5	33.5	±	12.3	19.8	±	5.32	14.1	±	4.28	14.5	±	3.68
	% of total FA	0.37	±	0.08	0.73	±	0.38	0.89	±	0.55	1.41	±	0.53	1.06	±	0.30	0.77	±	0.11	0.63	±	0.08	0.50	±	0.06
**C19:0**	μg/mL	1.01	±	0.32	1.10	±	0.31	1.29	±	0.31	1.55	±	0.34	1.14	±	0.31	0.95	±	0.22	0.92	±	0.26	1.31	±	0.31
	% of total FA	0.04	±	0.02	0.04	±	0.01	0.04	±	0.01	0.04	±	0.01	0.04	±	0.01	0.04	±	0.01	0.04	±	0.01	0.05	±	0.01
**C18:3n3**	μg/mL	15.2	±	4.96	45.8	±	30.5	72.2	±	60.7	123	±	69.4	72.1	±	29.7	28.7	±	8.77	14.7	±	3.97	19.6	±	5.70
	% of total FA	0.52	±	0.07	1.61	±	1.16	2.24	±	1.79	3.34	±	1.31	2.26	±	0.65	1.11	±	0.29	0.66	±	0.09	0.68	±	0.12
**C18:4n3**	μg/mL	<LLOQ			12.3	±	11.6	21.2	±	22.2	43.6	±	29.4	19.1	±	12.0	3.05	±	1.17	1.18	±	0.47	1.04	±	0.52
	% of total FA	<LLOQ			0.44	±	0.45	0.66	±	0.66	1.17	±	0.60	0.59	±	0.30	0.12	±	0.05	0.05	±	0.02	0.03	±	0.02
**C20:0**	μg/mL	5.62	±	1.04	5.77	±	1.14	6.28	±	1.41	6.63	±	1.79	6.49	±	1.34	6.04	±	1.13	4.92	±	0.94	7.37	±	1.08
	% of total FA	0.20	±	0.03	0.21	±	0.04	0.20	±	0.03	0.19	±	0.03	0.21	±	0.03	0.24	±	0.03	0.23	±	0.02	0.26	±	0.03
**C20:1n9**	μg/mL	7.17	±	2.54	7.28	±	2.14	7.12	±	2.81	7.77	±	3.25	6.01	±	1.82	5.26	±	1.35	4.03	±	0.78	5.12	±	1.31
	% of total FA	0.26	±	0.10	0.25	±	0.05	0.22	±	0.05	0.21	±	0.05	0.19	±	0.05	0.21	±	0.06	0.19	±	0.04	0.18	±	0.04
**C20:2n6**	μg/mL	6.04	±	1.69	6.13	±	1.75	6.83	±	1.81	6.59	±	1.44	6.32	±	1.07	5.42	±	1.23	4.65	±	1.08	6.31	±	1.29
	% of total FA	0.21	±	0.05	0.21	±	0.04	0.21	±	0.04	0.19	±	0.03	0.20	±	0.03	0.21	±	0.03	0.21	±	0.05	0.22	±	0.04
**C20:3n9**	μg/mL	4.93	±	2.73	4.90	±	2.76	5.41	±	3.11	5.06	±	2.57	5.45	±	2.34	4.39	±	2.24	3.42	±	1.51	4.43	±	1.67
	% of total FA	0.16	±	0.06	0.16	±	0.05	0.16	±	0.05	0.14	±	0.05	0.17	±	0.06	0.16	±	0.05	0.15	±	0.04	0.15	±	0.04
**C20:3n6**	μg/mL	50.2	±	12.4	48.7	±	14.1	54.3	±	14.1	55.0	±	12.5	63.3	±	11.8	60.6	±	12.9	50.1	±	10.4	70.1	±	15.4
	% of total FA	1.75	±	0.28	1.69	±	0.24	1.70	±	0.31	1.58	±	0.32	2.05	±	0.37	2.37	±	0.39	2.30	±	0.37	2.44	±	0.39
**C21:0**	μg/mL	<LLOQ			<LLOQ			<LLOQ			<LLOQ			<LLOQ			<LLOQ			<LLOQ			<LLOQ		
	% of total FA	<LLOQ			<LLOQ			<LLOQ			<LLOQ			<LLOQ			<LLOQ			<LLOQ			<LLOQ		
**C20:4n6**	μg/mL	182	±	53.1	175	±	62.8	194	±	67.1	195	±	59.7	225	±	64.6	189	±	59.5	159	±	44.2	224	±	55.7
	% of total FA	6.34	±	1.39	6.11	±	1.61	6.03	±	1.58	5.49	±	1.28	7.09	±	1.39	7.30	±	1.55	7.18	±	1.20	7.73	±	1.25
**C20:3n3**	μg/mL	<LLOQ			<LLOQ			0.65	±	0.18	0.99	±	0.29	0.64	±	0.23	0.68	±	0.27	<LLOQ			<LLOQ		

	% of total FA	<LLOQ	<LLOQ	0.02 ± 0.01	0.03 ± 0.01	0.01 ± 0.01	0.02 ± 0.02	<LLOQ	<LLOQ
**C20:4n3**	µg/mL	2.76 ± 1.36	3.41 ± 1.37	5.60 ± 2.52	9.39 ± 3.38	12.6 ± 3.95	8.86 ± 3.14	5.37 ± 1.97	6.44 ± 2.13
	% of total FA	0.09 ± 0.03	0.12 ± 0.04	0.18 ± 0.10	0.27 ± 0.11	0.40 ± 0.09	0.34 ± 0.09	0.24 ± 0.07	0.22 ± 0.06
**C20:5n3**	µg/mL	15.2 ± 6.57	15.2 ± 6.73	17.1 ± 7.88	18.2 ± 7.41	21.4 ± 8.19	22.5 ± 8.68	19.3 ± 7.39	24.8 ± 8.33
	% of total FA	0.52 ± 0.18	0.52 ± 0.17	0.52 ± 0.15	0.51 ± 0.16	0.66 ± 0.21	0.86 ± 0.27	0.85 ± 0.25	0.85 ± 0.23
**C22:0**	µg/mL	19.1 ± 3.17	19.7 ± 3.20	19.2 ± 3.99	17.5 ± 3.35	23.5 ± 4.51	19.2 ± 3.42	16.4 ± 3.10	22.4 ± 3.10
	% of total FA	0.68 ± 0.13	0.73 ± 0.24	0.62 ± 0.14	0.51 ± 0.11	0.76 ± 0.14	0.77 ± 0.16	0.75 ± 0.09	0.79 ± 0.09
**C22:1n9**	µg/mL	2.27 ± 1.01	4.82 ± 3.05	3.30 ± 3.30	3.85 ± 1.47	4.14 ± 2.06	2.28 ± 0.64	2.81 ± 2.52	3.93 ± 2.37
	% of total FA	0.08 ± 0.04	0.18 ± 0.13	0.11 ± 0.10	0.12 ± 0.06	0.14 ± 0.07	0.09 ± 0.03	0.13 ± 0.14	0.15 ± 0.10
**C22:2n6**	µg/mL	<LLOQ	<LLOQ	<LLOQ	<LLOQ	<LLOQ	<LLOQ	<LLOQ	<LLOQ
	% of total FA	<LLOQ	<LLOQ	<LLOQ	<LLOQ	<LLOQ	<LLOQ	<LLOQ	<LLOQ
**C22:4n6**	µg/mL	7.25 ± 1.92	7.39 ± 2.15	8.40 ± 2.54	7.52 ± 1.86	8.90 ± 2.12	7.39 ± 1.82	6.10 ± 1.27	9.19 ± 1.58
	% of total FA	0.25 ± 0.03	0.26 ± 0.04	0.26 ± 0.04	0.21 ± 0.03	0.28 ± 0.05	0.29 ± 0.03	0.28 ± 0.03	0.32 ± 0.03
**C22:5n6**	µg/mL	3.96 ± 1.81	3.90 ± 2.20	4.61 ± 1.81	4.44 ± 1.63	4.99 ± 1.75	4.05 ± 1.70	3.14 ± 1.20	4.49 ± 2.04
	% of total FA	0.13 ± 0.06	0.12 ± 0.07	0.14 ± 0.04	0.12 ± 0.03	0.16 ± 0.04	0.15 ± 0.04	0.14 ± 0.03	0.15 ± 0.05
**C22:5n3**	µg/mL	16.4 ± 6.27	13.5 ± 6.22	18.6 ± 5.13	18.7 ± 5.04	21.7 ± 5.84	17.6 ± 4.63	15.9 ± 4.18	24.1 ± 4.89
	% of total FA	0.56 ± 0.17	0.45 ± 0.15	0.59 ± 0.16	0.53 ± 0.11	0.69 ± 0.11	0.69 ± 0.15	0.72 ± 0.14	0.85 ± 0.15
**C24:0**	µg/mL	15.4 ± 2.85	14.14 ± 2.18	14.5 ± 3.04	13.1 ± 2.47	18.2 ± 4.03	16.0 ± 2.53	13.0 ± 2.12	18.0 ± 2.66
	% of total FA	0.54 ± 0.08	0.52 ± 0.14	0.47 ± 0.10	0.38 ± 0.09	0.59 ± 0.10	0.63 ± 0.11	0.60 ± 0.09	0.63 ± 0.09
**C22:6n3**	µg/mL	40.3 ± 12.4	39.8 ± 15.0	45.1 ± 16.4	42.7 ± 13.9	48.2 ± 15.1	39.4 ± 11.9	33.1 ± 9.84	48.7 ± 13.3
	% of total FA	1.38 ± 0.21	1.36 ± 0.27	1.37 ± 0.21	1.18 ± 0.18	1.51 ± 0.23	1.51 ± 0.22	1.49 ± 0.21	1.67 ± 0.22
**C24:1n9**	µg/mL	27.8 ± 4.57	26.6 ± 6.54	26.9 ± 6.26	26.6 ± 4.87	31.9 ± 7.29	30.1 ± 5.90	24.1 ± 4.31	35.8 ± 5.25
	% of total FA	1.00 ± 0.22	0.95 ± 0.24	0.88 ± 0.25	0.77 ± 0.17	1.03 ± 0.21	1.19 ± 0.21	1.11 ± 0.15	1.26 ± 0.16
**ΣFA**	µg/mL	2890 ± 691	2910 ± 885	3256 ± 944	3598 ± 1021	3161 ± 700	2584 ± 575	2198 ± 455	2881 ± 526
	% of total FA	100 ± 0.00	100 ± 0.00	100 ± 0.00	100 ± 0.00	100 ± 0.00	100 ± 0.00	100 ± 0.00	100 ± 0.00
**ΣSFA**	µg/mL	950 ± 249	978 ± 334	1100 ± 373	1238 ± 393	1043 ± 259	854 ± 212	748 ± 164	963 ± 199
	% of total FA	32.7 ± 1.53	33.3 ± 1.70	33.4 ± 2.12	34.2 ± 1.58	32.8 ± 1.51	32.9 ± 1.53	34.0 ± 1.20	33.3 ± 1.35
**ΣMUFA**	µg/mL	878 ± 283	868 ± 320	943 ± 336	981 ± 334	785 ± 208	674 ± 183	548 ± 131	727 ± 136
	% of total FA	30.0 ± 3.18	29.3 ± 3.05	28.5 ± 2.91	26.9 ± 2.53	24.6 ± 2.17	25.9 ± 2.01	24.9 ± 2.18	25.3 ± 1.82

**ΣPUFA**	µg/mL	1062	±	200	1064	±	258	1213	±	273	1379	±	321	1333	±	256	1056	±	208	902	±	177	1191	±	210
	% of total FA	37.3	±	3.79	37.4	±	4.33	38.1	±	4.32	39.0	±	3.61	42.5	±	2.73	41.2	±	3.02	41.1	±	1.80	41.4	±	1.99
**Σn3 PUFA**	µg/mL	90.4	±	28.7	130	±	54.1	180	±	90.3	257	±	114	196	±	64.3	121	±	34.0	89.6	±	25.7	125	±	31.6
	% of total FA	3.09	±	0.40	4.50	±	1.49	5.57	±	2.35	7.03	±	1.77	6.12	±	1.08	4.65	±	0.82	4.02	±	0.56	4.31	±	0.55
**Σn6 PUFA**	µg/mL	966	±	175	929	±	220	1027	±	221	1117	±	230	1132	±	208	931	±	180	809	±	153	1062	±	183
	% of total FA	34.0	±	3.83	32.7	±	4.19	32.3	±	3.92	31.8	±	3.97	36.2	±	3.15	36.3	±	2.92	37.0	±	1.95	37.0	±	2.08

Levels are shown as concentrations [µg/mL] and as relative concentrations [%] of total fatty acids at baseline (t0), and 2 (t2), 4 (t4), 6 (t6), 8 (t8), 24 (t24), 48 (t48), 72 (t72) hours after single-dose ingestion of echium oil (26 g). All data are shown as mean ± standard deviation (SD). FA, fatty acids; MUFA, monounsaturated fatty acids; n3, omega-3; n6, omega-6; PUFA, polyunsaturated fatty acids; SD, standard deviation; SFA, saturated fatty acids. In plasma, the minor fatty acid C12:0 was not evaluated due to chromatographic interferences.
